# Explication of the Potential of 2-Hydroxy-4-Methoxybenzaldehyde in Hampering Uropathogenic *Proteus mirabilis* Crystalline Biofilm and Virulence

**DOI:** 10.3389/fmicb.2019.02804

**Published:** 2019-12-10

**Authors:** Ravindran Durgadevi, Gurusamy Abirami, Rajaiah Alexpandi, Kumar Nandhini, Ponnuchamy Kumar, Santhiyagu Prakash, Arumugam Veera Ravi

**Affiliations:** ^1^Department of Biotechnology, Alagappa University, Karaikudi, India; ^2^Food Chemistry and Molecular Cancer Biology Lab, Department of Animal Health and Management, Alagappa University, Karaikudi, India; ^3^Department of Basic Science, Tamilnadu Dr. J. Jayalalithaa Fisheries University, Chennai, India

**Keywords:** 2-hydroxy-4-methoxybenzaldehyde, antivirulence, biofilm inhibition, crystalline biofilm, *Proteus mirabilis*

## Abstract

*Proteus mirabilis* is an important etiological agent of catheter-associated urinary tract infections (CAUTIs) owing to its efficient crystalline biofilm formation and virulence enzyme production. Hence, the present study explicated the antibiofilm and antivirulence efficacies of 2-hydroxy-4-methoxybenzaldehyde (HMB) against *P. mirabilis* in a non-bactericidal manner. HMB showed concentration-dependent biofilm inhibition, which was also evinced in light, confocal, and scanning electron microscopic (SEM) analyses. The other virulence factors such as urease, hemolysin, siderophores, and extracellular polymeric substances production as well as swimming and swarming motility were also inhibited by HMB treatment. Further, HMB treatment effectively reduced the struvite/apatite production as well as crystalline biofilm formation by *P. mirabilis*. Furthermore, the results of gene expression analysis unveiled the ability of HMB to impair the expression level of virulence genes such as *flhB*, *flhD*, *rsbA*, *speA*, *ureR*, *hpmA*, and *hpmB*, which was found to be in correlation with the results of *in vitro* bioassays. Additionally, the cytotoxicity analysis divulged the innocuous characteristic of HMB against human embryonic kidney cells. Thus, the present study reports the potency of HMB to act as a promising therapeutic remedy for *P. mirabilis*-instigated CAUTIs.

## Introduction

Urinary tract infections (UTIs) are the most prevalent bacterial infections acquired in health-care settings that can affect any part of urinary system including the kidneys, bladder, ureters, and urethra (Stamm and Norrby, 2001). Among the hospital-acquired UTIs, 70–80% are associated with the urinary catheter, which is a urine drainage tube inserted into the bladder through the urethra. The prolonged usage of indwelling urinary catheters is the most crucial risk factor for triggering complicated CAUTIs (Trautner, 2010). Bacterial pathogens are major contributors of complicated UTIs owing to their virulence secretion and biofilm formation on catheter surfaces (Soto, 2014; Flores-Mireles et al., 2015). In this midst, *Proteus mirabilis* is an imperious etiological agent of CAUTIs in patients undertaking long-term catheterization (Jacobsen et al., 2008).

*Proteus mirabilis*, a species of Morganellaceae family, is well known for its bulls’-eye-pattern swarming motility and urease activity (Schaffer and Pearson, 2015; Adeolu et al., 2016; Hamilton et al., 2018). The production of virulence factors aids in promoting the adhesion and biofilm formation of *P. mirabilis* on biological as well as abiotic urinary catheter surfaces (Hola et al., 2012). Owing to the flagella-mediated motility, *P. mirabilis* can easily transmit the infection to the urinary bladder through the urethra and to other parts of the host system through the bloodstream. During swarming, *P. mirabilis* co-regulates the expression of flagellar gene hierarchy as well as virulence genes (Belas and Suvanasuthi, 2005) encoding hemolysin HpmA and urease enzymes (Fraser et al., 2002; Morgenstein et al., 2010). These virulence factors are responsible for the complications of *P. mirabilis* infections including urolithiasis, bacteremia, and sepsis (Foxman and Brown, 2003).

Also, *P. mirabilis* has the ability to form crystalline biofilm under urinary environment, which happens as a sequel of alkalization of urine by the enzyme urease that catalyzes the hydrolysis of urea in urine and produces ammonia. Consequently, the production of ammonia leads to the precipitation of calcium and magnesium ions and formation of urinary stones composed of magnesium ammonium phosphate (struvite) and calcium phosphate (apatite). These precipitated minerals entrap within the *P. mirabilis* biofilm matrix formed on the urinary catheter, thus forming a dense crystalline biofilm and eventually blocking the urine flow through the catheter (Holling et al., 2014; Torzewska and Rozalski, 2014).

Moreover, the biofilm formation aids in protecting the pathogen from host defense mechanisms and boosts its resistance against antimicrobial drugs (Armbruster et al., 2018; Prywer et al., 2018). The sustained development of drug resistance in *P. mirabilis* sets up a severe menace in clinical settings. Therefore, prevention and appropriate treatment strategies are of utmost necessity. Research on plant resource-based traditional therapies has been tremendously increased worldwide for controlling human infectious diseases. In parallel, antibiofilm and antivirulence strategies have become an effective method to combat antimicrobial resistance (Cheng, 2016; Raorane et al., 2019). Inactivation of virulence secretion by targeting the transcriptional gene regulatory system will disarm the pathogens rather than killing or inhibiting their growth and will render them susceptible to natural host defenses. With due recognition of the global transcriptional regulator of virulence factor expression in bacterial pathogenicity, targeting such mechanism would be a precise way to control the bacterial infections (Jiang et al., 2019). As bactericidal activity of antibiotics exerts selection pressure on bacterial population, selective mutations are created in the population that allows the bacteria to escape drug activity (Totsika, 2017). Therefore, inhibition of virulence factors presents an attractive alternative to antibiotics. The virulence inhibitors may interfere with quorum signaling molecule, which regulates the expression of virulence factors and, thereby, abolishes bacterial pathogenicity (Yang et al., 2012).

Several prior studies have reported the proficient antibiofilm and antipathogenic properties of medicinal plants and their bioactive compounds against various clinically relevant bacterial pathogens (Harjai et al., 2010; Torzewska and Rozalski, 2014; Ramanathan et al., 2018; Alexpandi et al., 2019). Among the identified phytocompounds, 2-hydroxy-4-methoxybenzaldehyde (HMB), which is majorly present in the roots of traditional Indian plant, *Hemidesmus indicus*, has drawn the attention of researchers owing to its numerous biological properties (Wang et al., 2010; Srikanta et al., 2011; Kannappan et al., 2019). Although several previous reports have demonstrated the pharmacological activities of HMB, none of the studies have described its antibiofilm and antivirulence efficacies against Gram-negative bacterial pathogens. In light of the aforesaid facts, the present study unwinds the antibiofilm and antivirulence potential of HMB against the exclusive uropathogen, *P. mirabilis*.

## Materials and Methods

### Ethics Statement

In this study, blood samples were collected from healthy human volunteers by a trained technical person for blood survivability assay. The usage of human blood sample and experimental procedure was assessed and approved by the Institutional Ethical Committee, Alagappa University, under no. IEC/AU/2018/6.

### Bacterial Strain and Culture Condition

*Proteus mirabilis* reference strain (American Type Culture Collection [ATCC] 7002) and clinical isolate (CI) (GenBank accession no. MG905628) were used in this study. Both the strains were maintained in Luria–Bertani (LB; HiMedia, Mumbai) (pH 7.2 ± 0.2) agar plate and cultivated in LB broth at 120 rpm in a shaker overnight at 37°C. For experimental purpose, the overnight culture was subcultured in fresh LB broth until it reached 0.4 OD at 600 nm (10^8^ CFU/ml).

### 2-Hydroxy-4-Methoxybenzaldehyde

2-Hydroxy-4-methoxybenzaldehyde was purchased from Sigma-Aldrich (St. Louis, MO, United States). For experimental purpose, a stock solution of 50 mg/ml concentration of HMB was prepared using methanol.

### Determination of Minimum Inhibitory Concentration and Minimum Biofilm Inhibitory Concentration

The effect of HMB on *P. mirabilis* growth and biofilm formation was assessed by microbroth dilution method as described by Clinical and Laboratory Standards Institute [CLSI], 2016. Briefly, 1% of *P. mirabilis* (10^8^ CFU/ml) culture was added to LB media (1 ml) supplemented with increasing concentrations of HMB (25–400 μg/ml) and incubated at 37°C for 24 h. LB containing 0.8% of methanol and 1% of culture was considered as negative control. After incubation, the growth inhibition was measured at OD_600 nm_ using UV-Vis Spectrophotometer (U-2800, Hitachi, Japan). Minimum inhibitory concentration (MIC) was determined at the concentration of HMB that yields complete visible growth inhibition. Afterward, the planktonic cells were removed from both HMB-treated and untreated *P. mirabilis* samples, and the biofilm biomass was quantified by crystal violet (CV) staining method (Musthafa et al., 2010). The percentage of biofilm biomass inhibition was calculated using the following equation:% of Inhibition = ([Control OD_570 nm_ - Treated OD_570 nm_]/Control OD_570 nm_) × 100.

### Assessment of Cellular Viability Using 2,3-Bis(2-Methoxy-4-Nitro-5-Sulfophenyl)-2*H*-Tetrazolium-5-Carboxanilide Assay

The cellular activity of *P. mirabilis* under HMB treatment was measured by XTT [2,3-bis(2-methoxy-4-nitro-5-sulfophenyl)-2*H*-tetrazolium-5-carboxanilide] reduction assay. In the presence of an electron-coupling reagent (menadione), the cleavage of tetrazolium salt XTT produces a strongly colored soluble formazan salt through a complex cellular mechanism of metabolically active cells (Stockert et al., 2018). In the present study, the HMB-treated and untreated *P. mirabilis* cells were incubated with the XTT–menadione mixture [50 μl of XTT (Sigma); 4 μl of menadione (HiMedia, Mumbai)] for 1 h at 37°C in the dark. Following incubation, the absorbance of formazan dye formed in the cell-free supernatants was measured at OD_470 nm,_ which directly correlates to the number of viable cells in the culture (Singh et al., 2011).

### Microscopic Analysis

*Proteus mirabilis* was allowed to form biofilm on the surface of glass slides (1 × 1 cm) in the absence and presence of HMB [minimum biofilm inhibitory concentration (MBIC)] and incubated at 37°C for 24 h. After incubation, the slides were rinsed with distilled water and dried in air. Then the slides were stained using 0.4% CV or 0.1% acridine orange (AO) for light microscopic (LM) (Nikon Eclipse 80i, Shinagawa-ku, Japan) and confocal laser scanning microscopic (CLSM) (Zeiss LSM 710, Germany) analyses, respectively.

For SEM analysis, HMB-treated and untreated biofilm cells that formed on the glass slides were fixed using glutaraldehyde (2.5%) at 4°C for 2 h. Then the slides were gradually dehydrated using increasing concentrations (20, 40, 60, 80, and 100%) of ethanol. After that, the gold prior sputter coating was implemented in the slides to be observed under the field-emission SEM (FESEM) (Quanta FEG 200).

### Effect of 2-Hydroxy-4- Methoxybenzaldehyde on *Proteus mirabilis* Exopolysaccharide Production

#### Exopolysaccharide Extraction and Quantification

The cell-free and cell-bound exopolysaccharides (EPSs) were extracted from HMB-treated and untreated *P. mirabilis* samples as described by Sivasankar et al. (2016) with minor modifications. Briefly, *P. mirabilis* cells were grown at 37°C for 24 h in LB media supplemented with and without HMB (MBIC). After incubation, the cell-free culture supernatant (CFCS) was collected from both HMB-treated and untreated samples by centrifugation at 8,000 rpm for 10 min. Subsequently, the cell pellets were suspended in freshly prepared isotonic buffer (pH 8.0 ± 0.2), which contains Tris (10 mM), EDTA (10 mM), and NaCl (2.5%) and incubated overnight at 4°C. After incubation, supernatant containing the cell-bound EPS was collected and pooled with CFCS. Then ice-cold ethanol was added with the aforesaid supernatant mixture at 2:1 ratio for precipitation. The precipitated cell-free and cell-bound EPS was collected by centrifugation at 12,000 rpm for 20 min. Finally, the extracted EPS samples were weighed, and% of EPS reduction was calculated using the formula (Control EPS wt - Treated EPS wt/Control EPS wt) × 100.

#### Fourier Transform Infrared Spectroscopy Analysis

For Fourier transform infrared (FT-IR) analysis, an equal quantity of EPS extracted from HMB-treated and untreated *P. mirabilis* was pelletized separately with potassium bromide (KBr) at 1:100 ratio under hydraulic pressure. The IR spectra of EPS samples were scanned in the spectral range of 2,000–500 cm^–1^ using FT-IR spectroscopy (Nicolet iS5, Thermo Fisher Scientific Inc., Canton, GA, United States). The spectra were plotted as absorbance versus wavenumber and analyzed for variations in EPS components using OMNIC software.

### Effect of 2-Hydroxy-4- Methoxybenzaldehyde on the Virulence Factors of *Proteus mirabilis*

#### Urease Assay

The effect of HMB on *P. mirabilis* urease production was determined by the procedure described Durgadevi et al. (2019) with modification. Briefly, 1% of *P. mirabilis* culture was allowed to grow in 1 ml of urea broth [peptone (1.5 g/L), sodium chloride (5 g/L), monopotassium phosphate (2 g/L), and filter sterilized urea (20 g/L)] supplemented with and without HMB (12.5, 25, and 50 μg/ml) for 24 h at 37°C. After incubation, 0.02% phenol red reagent (pH indicator) was added to CFCS of both HMB-treated and untreated samples. Owing to the urease activity, the pH of the samples increases, which is indicated by the formation of pink color. Then, the color change was visually observed and spectroscopically measured at OD_670 nm._

#### Hemolysin Assay

The effect of HMB on *P. mirabilis* hemolysin production was assessed by following the method of Subramenium et al. (2015). Briefly, an equal volume of CFCS of both HMB-treated (12.5, 25, and 50 μg/ml) and untreated samples was incubated with 2% of freshly collected sheep blood (in sterile phosphate-buffered saline [PBS] [v/v]) and incubated at 37°C for 1 h. During the incubation period, the hemolysin enzyme lyses the red blood cells by destroying their cell membrane. And the released blood cell components were mixed with the sample, and its density was increased. Then, the cell-free supernatants were collected by centrifugation at 3,000 rpm for 5 min and spectroscopically measured at OD_405 nm_ in order to quantify the hemolysin production.

#### Siderophore Assay

The effect of HMB on siderophore production by *P. mirabilis* was assessed by blue agar chrome azurol sulfonate (CAS) assay. CAS agar was prepared by a step-by-step procedure as described by Louden et al. (2011) with minor modification. Briefly, 0.3% of piperazine-*N*,*N*′-bis(2-ethanesulfonic acid) (PIPES; HiMedia, Mumbai) was added to 10 ml of Minimal Media 9 (MM9) salt solution (3% KH_2_PO_4_, 10% NH_4_Cl, and 5% NaCl). Then, seven volumes of ddH_2_O and 2% of agar were added to the MM9/PIPES mixture, and its pH was adjusted to 6.8 using 0.1 N of NaOH solution. Simultaneously, blue dye was prepared by pooling the three individual solutions including 0.1% CAS, 0.03% FeCl_3_, and 0.2% hexadecyl trimethyl ammonium bromide (HiMedia, Mumbai) at 5:1:4 ratio. The prepared MM9/PIPES and blue dye solutions were autoclaved separately and cooled to 50°C. The CAS agar was prepared by pooling the filter sterilized tryptone (10%; HiMedia, Mumbai) and glucose (20%; HiMedia, Mumbai) solutions with MM9/PIPES mixture. Finally, the blue dye was gradually added to the CAS agar and mixed thoroughly. The prepared CAS agar was supplemented with and without of HMB (MBIC) and poured aseptically into sterile petri plates. Then, 2 μl of bacterial culture was placed at the center of these CAS agar plates. After incubation for a period of 24 h, the plates were analyzed for the zone of siderophore activity.

### Crystalline Biofilm Production

Owing to urease activity, *P. mirabilis* produces crystalline biofilm in the presence of urinary components such as urea, chloride, sodium, potassium, and other dissolved ions, and organic and inorganic compounds. For this study, artificial urine (AU) media was prepared in accordance with the procedure described by Durgadevi et al. (2019) with minor changes. Briefly, 0.05% calcium chloride, 0.07% magnesium chloride, 0.2% sodium disulfate, 0.5% sodium chloride, 0.002% sodium oxalate, 0.06% trisodium citrate, 0.1% ammonium chloride, 0.3% potassium dihydrogen orthophosphate, 0.2% potassium chloride, and 2.5% urea were dissolved in sterile distilled water. Then, the pH of the prepared AU media was adjusted to 6.2 ± 0.2 and sterilized using a cellulose nitrate membrane filter (0.2 μm; Sartorius, United Kingdom). Finally, the filtered AU medium was aseptically pooled with equal volume of sterile 2 × LB medium for experimental purposes.

### Characterization Analysis

*Proteus mirabilis* culture was allowed to grow in AU media at 37°C for 24 h. After incubation, the planktonic cells were discarded, and the crystal precipitates (due to generation of alkaline environment) were collected and dried by vacuum drier. Further, the qualitative analysis of the precipitated struvite/apatite crystals was carried out by X-ray diffraction (XRD) spectrophotometer (X’pert Pro; PANalytical, Almelo, Netherlands) and FT-IR spectroscopy (Nicolet iS5, Thermo Fisher Scientific Inc., Canton, GA, United States) to determine its degree of crystallization as well as the presence of inorganic chemical composition.

### Crystalline Biofilm Inhibition Assay

The effect of HMB on *P. mirabilis* crystalline biofilm formation was assessed by microbroth dilution method as aforementioned. Briefly, 1% of *P. mirabilis* culture was allowed to grow in AU media supplemented with and without HMB (25, 50, and 100 μg/ml) for 24 h at 37°C. Then, the crystalline biofilm production in the presence and absence of HMB was quantified by CV staining method. In addition, the anti-crystalline biofilm efficacy of HMB against *P. mirabilis* was observed by LM and CLSM techniques. For this, the samples were prepared as mentioned above.

### Scanning Electron Microscopy and Energy-Dispersive X-Ray Analysis

To analyze the effect of HMB on *P. mirabilis* crystalline biofilm production, FESEM with energy-dispersive X-ray (EDAX) analysis was performed. *P. mirabilis* was allowed to grow on glass slides immersed in AU media supplemented with and without HMB (MBIC). Then the crystalline biofilm of control and treated slides were documented under SEM, and the level of struvite/apatite composition present in both samples was also examined by EDAX analysis.

### Survivability of *Proteus mirabilis* in Human Blood

The effect of HMB on cellular viability of *P. mirabilis* in blood serum was assessed by blood survival assay as described by Viszwapriya et al. (2016). *P. mirabilis* cell suspension was incubated with freshly collected human blood sample at 1:4 ratio in the absence and presence of HMB (at MBIC) for 3 h at 37°C. After incubation, HMB-treated and untreated blood samples were serially diluted and spread over LB agar plate to analyze the number of viable cells present in the HMB-treated and untreated samples.

### Motility Assessment

The effect of HMB on *P. mirabilis* swimming as well as swarming motility was analyzed as described by Liaw et al. (2000) with little modification. Initially, agar medium in the presence and absence of HMB (MBIC) was prepared for swarming and swimming motility assays separately. For assessing swarming motility, 3 μl of *P. mirabilis* culture was spot inoculated on the surface of LB agar plate (1% peptone, 0.5% NaCl, and 1.5% agar). For swimming motility, 5 μl of culture was stab inoculated at the center of semisolid LB agar plate (1% peptone, 0.5% NaCl, and 0.5% agar). The plates were then incubated at 37°C for 24 h with lid position facing upward.

### Live/Dead Staining Assay

Confocal laser scanning microscopic analysis was performed to evaluate the mature biofilm (24-h-old) disruption ability of HMB using live/dead staining (BacLight cell viability staining kit, Molecular Probes, Eugene, OR, United States). Briefly, *P. mirabilis* strains ATCC and CI were allowed to form biofilm on glass slides for 24 h at 37°C. Then, the planktonic cells were discarded, and the glass slide-bound biofilm was submerged in sterile 1 × PBS supplemented with and without HMB (1× and 2 × MBIC) and incubated for another 24 h. At the end of incubation, the slides were washed and stained with a mixture of propidium iodide (PI) and SYTO9 (1 μM of concentration each). The stained biofilm slides were documented under CLSM and examined with Zeiss LSM Image Browser (version 4.2.0.121).

### Quantitative Real-Time PCR Analysis

The total RNA was isolated from HMB-treated (MBIC) and untreated *P. mirabilis* by Trizol method. Then reverse transcription PCR was performed to convert the isolated RNA into cDNA using high capacity reverse transcriptase kit (Applied Biosystems Inc., United States). After that, quantitative real-time PCR (qRT-PCR) analysis was performed using SYBR Green PCR Master Mix in 7500 Sequence Detection System (Applied Biosystems Inc., United States). For qRT-PCR analysis, the expression of virulence genes (*flhB*, *flhD*, *rsbA*, *speA*, *ureR*, *hpmA*, and *hpmB*) was analyzed using gene-specific primers listed in Table 1. Then, the housekeeping gene *rplS* was used as an internal control to normalize the expression levels of the candidate genes (Ravindran et al., 2018).

**TABLE 1:** List of virulence genes in *P. mirabilis* and their primer sequences.

**Gene**	**Tm(°C)**	**Accession numbers**	**Primers**	
			
			**Forward primer**	**Reverse primer**
*flhB*	56	KGA90550	TCAGCTAACGCATTCATTG	GCCAGTGTTTCTAGGCTTG
*flhD*	56	TFU19979	CTTCCGCAATGTTTAGACTG	ATTTGTTGCAAATCATCCAC
*rsbA*	58	AAC82660	AAAATACAAGGCACTTTGACC	TATCAAGCTGTTGGCAGATTA
*speA*	57	QES78361	CATCTGTGATCCAAGGTGAA	GCCGAACGTTTAGAAGTGAT
*ureR*	56	CAA79243	GGATGTAGCAAAAACGCTCT	ATGCGTCACAAAAATAAGCA
*hpmA*	67	WP_149127624	GTTGAGGGGCGTTATCAAGAGTC	GATAACTGTTTTGCCCTTTTGTGC
*hpmB*	60	WP_143475422	CAGTGGATTAAGCGCAAATG	CCTTCAATACGTTCAACAAACC
*rplS*	58	TFU20016	CGAACAAGAACAAATGAAGC	GGGGAGTGAGTTTGGAATAC

### Assessment of Cytotoxicity of 2-Hydroxy-4-Methoxybenzaldehyde

The effect of HMB on human embryonic kidney cells (HEK-293) was assessed by MTT assay. Initially, 10^4^ cells/well of HEK-293 were seeded with Dulbecco’s modified Eagle medium (DMEM) (supplemented with 10% fetal bovine serum) in a 96-well microtiter plate and incubated in CO_2_ incubator at 37°C for 24 h. After incubation, the cells were exposed to different concentrations of HMB (25–500 μg/ml) supplemented in the medium and incubated for 24 h. Then, the medium was aspirated and added with 10 μl of MTT from 5 mg/ml stock (prepared in PBS) and incubated for an additional 3 h. The formazan crystals that formed at the end of incubation period were dissolved using DMSO, and the absorbance of the solution was measured at OD_590 nm_ in order to quantify the cytotoxicity of HMB.

Further, fluorescence microscopy analysis was performed to investigate the viability of HEK-293 cells under HMB (25–500 μg/ml) treatment. The HMB-treated and untreated cell lines were stained with a mixture of live/dead stains such as AO and ethidium bromide dyes (1:1 ratio), respectively. Then, the stained cells were imaged under inverted fluorescence microscope (Accu-Scope EXI-310).

### Statistics

In this study, all the *in vitro* experiments were performed in triplicate and repeated at least thrice. IBM SPSS statistics 23 (SPSS Ltd., Hong Kong) software package was used for statistical analysis. All data were analyzed using either one-way ANOVA or Student’s *T*-test for comparing the differences between control and treated samples.

## Results

### Antibacterial and Antibiofilm Activity of 2-Hydroxy-4-Methoxybenzaldehyde

**FIGURE 1:**
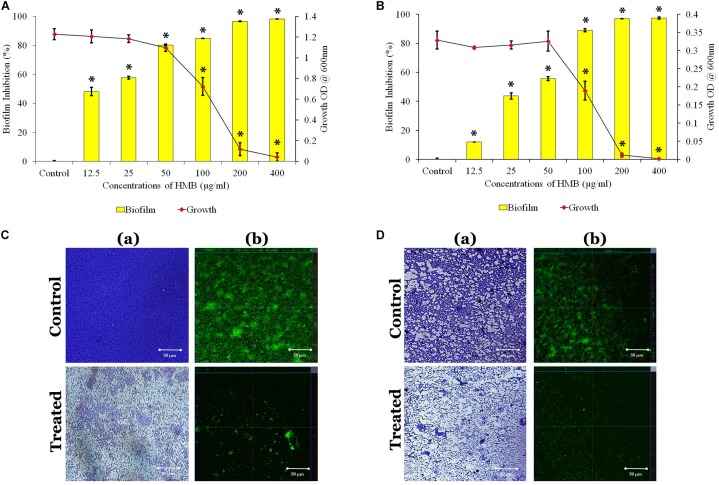
Effect of HMB (at its MBIC) on growth and biofilm of *Proteus mirabilis* **(A)** ATCC and **(B)** CI. Data represent mean values (*n* = 3) ± standard deviation. ^∗^ indicates significance at *P* < 0.005. Microscopic analysis of *P. mirabilis* **(C)** ATCC and **(D)** CI in the absence and presence of HMB at MBIC. (a) LM (400× magnification) and (b) CLSM (scale bar = 50 μm) images clearly portray a significant reduction in biofilm thickness of HMB (MBIC)-treated samples compared with untreated control. HMB, 2-hydroxy-4-methoxybenzaldehyde; MBIC, minimum biofilm inhibitory concentration; ATCC, American Type Culture Collection; CI, clinical isolate; LM, light microscopy; CLSM, confocal laser scanning microscopy.

The antibacterial activity of HMB against both *P. mirabilis* ATCC and CI was studied by broth dilution method. MIC was determined at 200 μg/ml concentration of HMB, wherein the visible growth of *P. mirabilis* strains was completely reduced (Figures 1A,B). Further, the antibiofilm efficacy of HMB was evaluated by biofilm biomass quantification method, and the results exhibited a concentration-dependent biofilm inhibition (Figures 1A,B). MBIC was determined as the minimum concentration of HMB that reduces the maximum biofilm production in *P. mirabilis*. MBIC of HMB was found to be 50 μg/ml, at which a maximum 80% of biofilm inhibition in *P. mirabilis* ATCC and 70% of biofilm inhibition in *P. mirabilis* CI were observed without any deleterious effect on the growth of planktonic cells. Thus, MBIC of HMB was used to carry out all virulence assays.

Further, the non-bactericidal antibiofilm property of HMB was validated by XTT reduction assay. The obtained result shows that the level of colored formazan production, indicating the presence of metabolic activity is the same in both HMB-treated and untreated culture. This observation concludes that the HMB (up to MBIC) treatment did not show any lethal effect on the cellular viability of *P. mirabilis* ATCC and CI (Supplementary Figure S1).

### Microscopic Analysis

The LM and CLSM analyses revealed the antibiofilm potential of HMB against *P. mirabilis* ATCC and CI strains wherein the biofilm architecture in HMB-treated slides was slackened and poorly formed than that of their respective untreated control slides (Figures 1C,D). Further, SEM analysis clearly portrayed that HMB effectually reduced biofilm-associated bacterial cells, whereas the control image showed an unimpaired and well-developed biofilm structure (Supplementary Figure S2).

### Effect of 2-Hydroxy-4- Methoxybenzaldehyde on Exopolysaccharide Production

**FIGURE 2:**
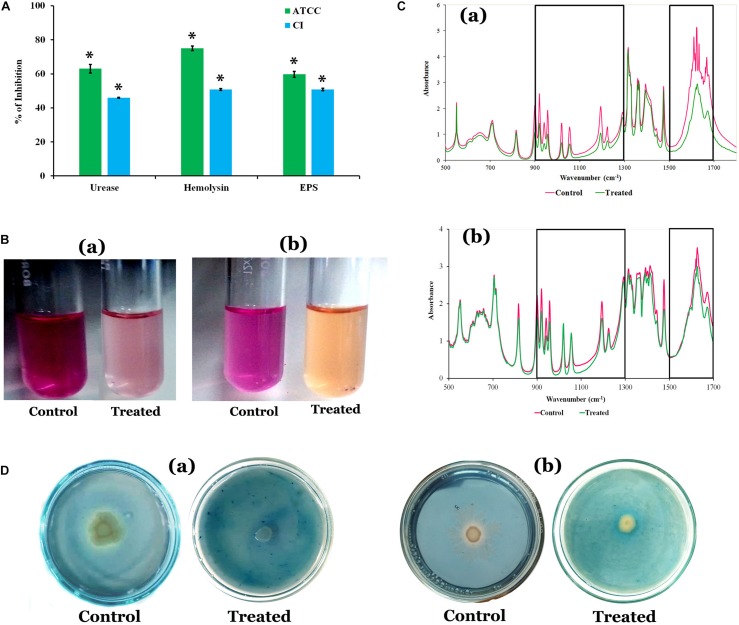
**(A)** Effect of HMB (at its MBIC) on *Proteus mirabilis* virulence factors such as urease, hemolysin, and EPS production. Data represent mean values (*n* = 3) ± standard deviation. ^∗^ indicates significance at *P* < 0.005. **(B)** Representative images of test tubes displaying urease inhibitory potential of HMB (MBIC) on *P. mirabilis* (a) ATCC and (b) CI. **(C)** FT-IR analysis of the EPS samples extracted from the HMB-treated (at its MBIC) and untreated *P. mirabilis* (a) ATCC and (b) CI. *Box*I represents the region of polysaccharides and nucleic acids (1,300–900 cm^–1^), and box II represents the region of proteins (1,500–1,700 cm^–1^). **(D)** Siderophore inhibitory potential of HMB at its MBIC on *P. mirabilis* (a) ATCC and (b) CI. HMB, 2-hydroxy-4-methoxybenzaldehyde; MBIC, minimum biofilm inhibitory concentration; EPS, exopolysaccharide; ATCC, American Type Culture Collection; CI, clinical isolate; FT-IR, Fourier transform infrared.

The quantification of EPS unveiled that HMB significantly reduced the EPS production. A maximum of 60 and 51% reduction was observed at MBIC of HMB treatment in *P. mirabilis* ATCC and CI, respectively (Figure 2A). In FT-IR analysis, the IR spectra of EPS samples extracted from the control and HMB-treated (MBIC) *P. mirabilis* culture exhibited indistinguishable peak positions at 1,500–1,700 and 1,300–900 cm^–1^, which corresponded to the proteins and mixed region of carbohydrates and nucleic acids, respectively (Durgadevi et al., 2019). However, the absorbance intensities of these spectral regions were found to be decreased in the EPS of HMB-treated *P. mirabilis* samples than those of the EPS of untreated control *P. mirabilis* samples (Figure 2C). This observation emphasizes that the production of EPS components in *P. mirabilis* were efficiently inhibited upon HMB treatment.

### Quantification of Urease and Hemolysin Production

The urease and hemolysin are the most important virulence factors produced by *P. mirabilis* to cause complicated CAUTIs. The results showed a maximum 63 and 46% of urease inhibition, and 75 and 51% of hemolysin inhibition upon treatment with HMB (MBIC) in *P. mirabilis* ATCC and CI, respectively (Figure 2A).

### Siderophore Inhibition

*Proteus mirabilis* produces iron-scavenging molecule siderophore, which is essential for its growth and biofilm development under iron-deficient conditions like human urinary tract system (Banin et al., 2005; Himpsl et al., 2010). Hence, attenuation of siderophores production could be considered as one of the possible ways to prevent *P. mirabilis*-mediated UTI. The HMB effectually inhibited the siderophore production in both ATCC and CI of *P. mirabilis* at its MBIC. A pale yellow zone of siderophore activity was observed around untreated *P. mirabilis* colonies in blue dye background, which has occurred via removal of iron content from the CAS, whereas in HMB-treated CAS agar plates, the zone of siderophore activity was found to have disappeared (Figure 2D).

### Crystalline Biofilm Formation by *Proteus mirabilis*

**FIGURE 3:**
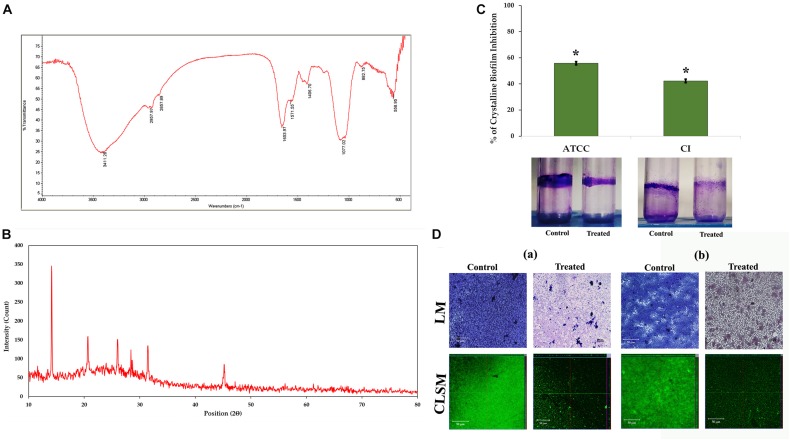
Crystalline biofilm production. **(A)** FT-IR and **(B)** XRD analyses of struvite/apatite crystal precipitates. **(C)** Assessment of HMB (at its MBIC) on crystalline biofilm of *Proteus mirabilis*. Data represent mean values (*n* = 3) ± standard deviation. ^∗^ indicates significance at *P* < 0.005. **(D)** Microscopic (LM and CLSM) analysis of *P. mirabilis* (a) ATCC and (b) CI crystalline biofilm formation in the presence and absence of HMB at MBIC. FT-IR, Fourier transform infrared; XRD, X-ray diffraction; HMB, 2-hydroxy-4-methoxybenzaldehyde; MBIC, minimum biofilm inhibitory concentration; LM, light microscopy; CLSM, confocal laser scanning microscopy; CI, clinical isolate.

The urease production by *P. mirabilis* causes obstruction and encrustation of urethral catheters via crystalline biofilm formation. Initially, owing to the urease activity of *P. mirabilis*, the local environmental pH greatly increases, which leads to the precipitation of soluble ions as struvite/apatite crystals in AU media. In this study, the FT-IR analysis was performed to confirm the production of struvite/apatite crystal precipitates. FT-IR spectrum of this salt precipitant shows high intensity IR bands at 3,411, 2,957, 2,857, 1,653, 1,571, 1,406, 1,077, 882, and 558 cm^–1^ regions, which corresponds to the presence of water of hydration, N–H bond, P–O bond, NH_4_^+^ ion and PO_4_^3–^ ion, and metal–oxygen bond. According to the previous report of Chauhan and Joshi (2013), the principal IR bands observed in FT-IR spectra of crystal precipitate are speculated to represent the presence of main components of struvite/apatite such as magnesium, ammonium, and calcium phosphates (Figure 3A).

Moreover, the crystallite nature of these salt precipitates was revealed by XRD analysis. Figure 3B shows sharp XRD peaks at 14.15°, 20.67°, 26.02°, 28.42°, 31.46°, and 45.57°, indicating the formation of well crystalline level of salt precipitants. The 2θ values were also found to be closely matching those of the previous report of Possenti et al. (2019), wherein the XRD patterns of struvite (peaks at 14.99°, 15.81°, 16.47°, 20.85°, 21.45°, 27.07°, and 31.94°) and apatite (peak at ∼25.90°) were depicted by comparing them with the Inorganic Crystal Structure Database (ICSD nos. 014269 and 016132). Altogether, the obtained results revealed the presence of magnesium ammonium phosphate and calcium phosphate crystals in the salt precipitates produced by *P. mirabilis*.

### Impact of 2-Hydroxy-4- Methoxybenzaldehyde on Crystalline Biofilm Production

The urease production by *P. mirabilis* causes obstruction and encrustation of urethral catheters via crystalline biofilm formation. Hence, the present study evaluates the potential of HMB on *P. mirabilis* crystalline biofilm production. HMB at MBIC exhibited 55 and 42% reduction of crystalline biofilm production in *P. mirabilis* ATCC and CI, respectively (Figure 3C). Further, the crystalline biofilm inhibitory potential of HMB was validated through LM and CLSM analyses wherein loosely attached microcolonies were observed in HMB-treated micrographs, whereas multilayered biofilm matrix was observed in the micrographs of untreated control samples of *P. mirabilis* ATCC and CI (Figure 3D).

### Scanning Electron Microscopy and Energy-Dispersive X-Ray Analysis

**FIGURE 4:**
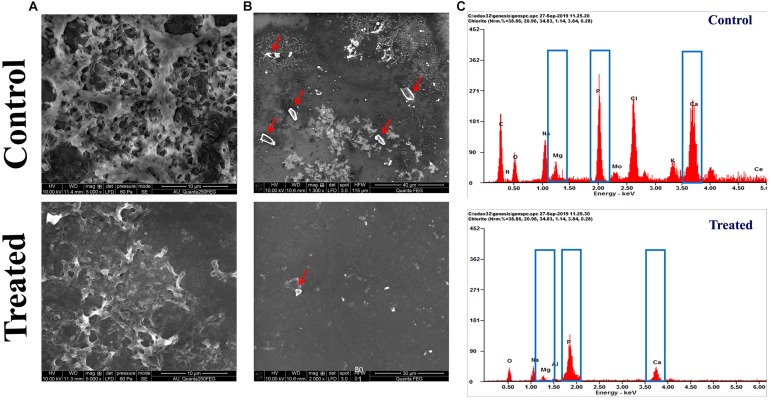
Effect of HMB (at its MBIC) on *Proteus mirabilis* crystalline biofilm production. FESEM analysis of **(A)** crystalline biofilm matrix and **(B)** formation of salt crystal precipitates (indicated by red arrow) and **(C)** energy-dispersive X-ray EDAX analysis of *P. mirabilis* struvite production in the presence and absence of HMB at MBIC. HMB, 2-hydroxy-4-methoxybenzaldehyde; MBIC, minimum biofilm inhibitory concentration; EDAX, energy-dispersive X-ray.

The FESEM analysis of *P. mirabilis* crystalline biofilm upon HMB treatment further validated the result of *in vitro* crystalline biofilm inhibition assay. In Figure 4A, the FESEM image of HMB-treated sample shows highly reduced crystalline biofilm formation, whereas the untreated control image shows densely formed crystalline biofilm matrix.

Also, the efficacy of HMB on the production of struvite/apatite salt crystals by *P. mirabilis* was evaluated by FESEM and EDAX analyses. Owing to the urease activity of *P. mirabilis*, soluble ions in urine will be precipitated normally as struvite/apatite. These precipitated crystals can grow to remarkable size and produce kidney stones as well as crystalline biofilm. In this study, the FESEM analysis revealed the struvite crystals as well as crystalline biofilm inhibitory efficacy of HMB. Figure 4B shows a high level of salt crystals in biofilm matrix of untreated control sample, whereas the HMB-treated sample shows only a low quantity of salt crystal precipitates. Meanwhile, EDAX analysis was performed to quantify the level of struvite components in the crystalline biofilm of *P. mirabilis* upon HMB treatment. In Figure 4C, it is seen that the peak intensity of crystallized struvite components such as calcium, magnesium, and phosphorus was highly reduced in HMB (MBIC)-treated sample in comparison with the untreated control.

### Effect of 2-Hydroxy-4- Methoxybenzaldehyde on *Proteus mirabilis* Viability in Human Blood

**FIGURE 5:**
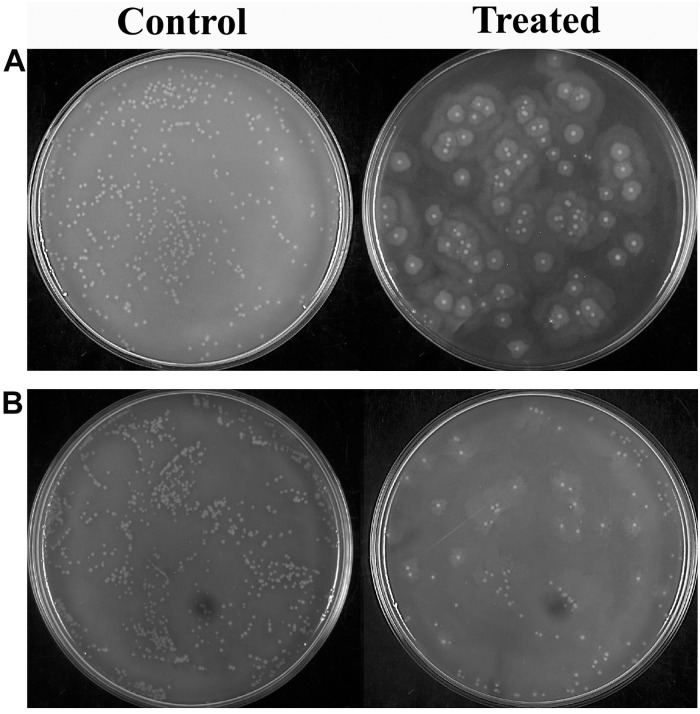
Effect of HMB on blood survivability of *Proteus mirabilis* **(A)** ATCC and **(B)** CI. Representative images clearly depict the efficient inhibition of blood survival rate of HMB (at its MBIC) treated sample when compared with control. HMB, 2-hydroxy-4-methoxybenzaldehyde; ATCC, American Type Culture Collection; CI, clinical isolate; MBIC, minimum biofilm inhibitory concentration.

*Proteus mirabilis* can potentially invade into the bloodstream from the upper urinary system, which further leads to septicemia. This is typically observed as the result of urinary catheterization or kidney infection. Thus, the efficacy of HMB treatment on cellular viability of *P. mirabilis* in human blood was assessed. HMB treatment efficiently reduced the total number of *P. mirabilis* cells in the blood sample than that of untreated control (Figure 5). Hence, the present study revealed that HMB enhances the susceptibility of *P. mirabilis* to human serum.

### Anti-swimming and Anti-swarming Activity of 2-Hydroxy-4- Methoxybenzaldehyde

**FIGURE 6:**
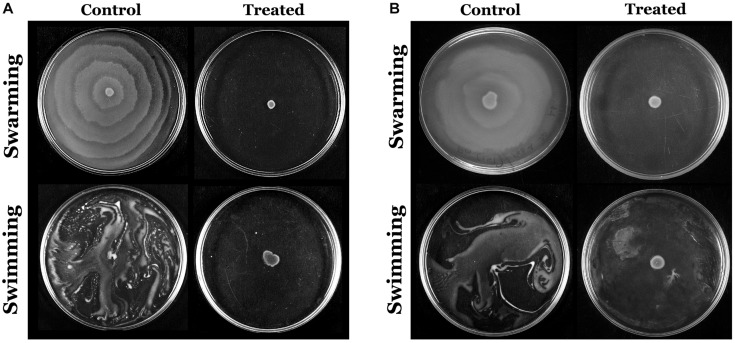
Assessment of inhibitory efficacy of HMB (at its MBIC) on swarming and swimming motility of *Proteus mirabilis* **(A)** ATCC and **(B)** CI. HMB, 2-hydroxy-4-methoxybenzaldehyde; MBIC, minimum biofilm inhibitory concentration; ATCC, American Type Culture Collection; CI, clinical isolate.

The flagella-mediated swimming and swarming motilities are the most important characteristic features of *P. mirabilis* pathogenicity, which facilitate the dissemination of the infection from urinary catheter to bladder and other parts of the host system (Jones et al., 2005). Hence, the efficacy of HMB on the motility of *P. mirabilis* was evaluated. The obtained results divulged that HMB (MBIC) effectually suppressed the swarming as well as swimming motilities in both ATCC and CI of *P. mirabilis*. In Figure 6, it is seen that the distance of bacterial motility from the inoculum point was highly reduced in the HMB-treated plates compared with their respective control plates.

### Effect of 2-Hydroxy-4- Methoxybenzaldehyde on *Proteus mirabilis* Preformed Biofilm

**FIGURE 7:**
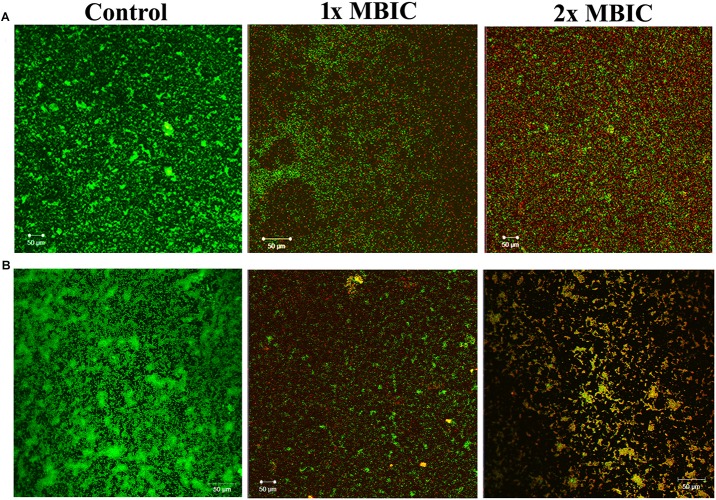
Confocal laser scanning microscopic analysis of the effect of HMB (at 1 × MBIC and 2 × MBIC) on preformed biofilm (24 h old) of *Proteus mirabilis* **(A)** ATCC and **(B)** CI using live/dead BacLight bacterial viability staining. Green fluorescence (SYTO9) represents viable cells and red fluorescence (PI) represents dead cells. CLSM, confocal laser scanning microscopy; HMB, 2-hydroxy-4-methoxybenzaldehyde; MBIC, minimum biofilm inhibitory concentration; ATCC, American Type Culture Collection; CI, clinical isolate; PI, propidium iodide.

The live/dead staining method was performed to divulge the killing efficacy of HMB against biofilm-associated cells of *P. mirabilis* ATCC and CI. The CLSM micrographs of HMB (1× and 2 × MBIC)-treated samples showed an elevated level of red fluorescence (PI), which indicates the dead cells, whereas the untreated control images displayed a high intensity of green fluorescence due to SYTO9, which specifically stains viable cells (Figure 7). This observation clearly revealed the concentration-dependent killing efficacy of HMB on biofilm-embedded *P. mirabilis* cells.

### Effect of 2-Hydroxy-4- Methoxybenzaldehyde on *Proteus mirabilis* Virulence Gene Expression

**FIGURE 8:**
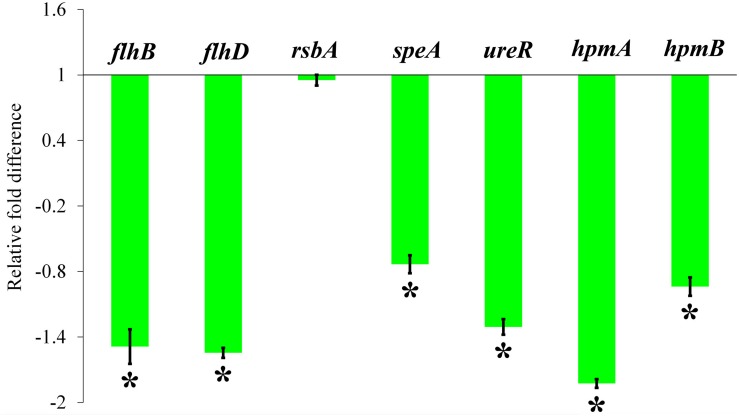
Effect of HMB on virulence genes expression in *Proteus mirabilis*. Bar chart depicting the relative fold difference in the expression level of virulence genes of *P. mirabilis* in the presence of HMB at MBIC. Data represent mean values (*n* = 3) ± standard deviation. ^∗^ indicates significance at *P* < 0.005. HMB, 2-hydroxy-4-methoxybenzaldehyde; MBIC, minimum biofilm inhibitory concentration.

The relative expression level of *P. mirabilis* virulence traits under HMB (MBIC) treatment was assessed by transcriptomic analysis. The obtained result exhibited that the genes *flhB*, *flhD*, *speA*, *ureR*, *hpmA*, and *hpmB* are significantly downregulated. Meanwhile, there was no significant relative fold change in *rsbA* gene upon HMB treatment (Figure 8).

### Efficacy of 2-Hydroxy-4- Methoxybenzaldehyde on HEK-293 Cells

**FIGURE 9:**
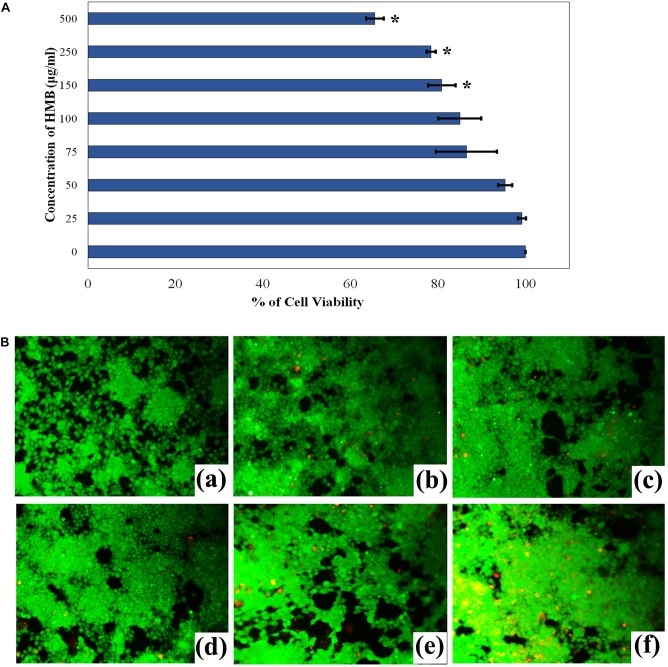
**(A)** Cytotoxicity assessment of HMB in HEK-293 cells by MTT assay. Data represent mean values (*n* = 3) ± standard deviation. ^∗^ indicates significance at *P* < 0.005. **(B)** Fluorescence microscopic images (200× magnifications) of control (a), 25 μg/ml (b), 50 μg/ml (c), 100 μg/ml (d), 250 μg/ml, (e) 500 μg/ml, and (f) of HMB-treated samples, which clearly depict the non-toxic nature of HMB. Green fluorescence represents viable cell lines, and red fluorescence represents dead cells. HMB, 2-hydroxy-4-methoxybenzaldehyde.

The non-cytotoxic nature of HMB on HEK-293 cell lines was analyzed by MTT assay. As the concentration of HMB increases, no obvious change was observed in the cell viability upon comparison with control (Figure 9A). To ensure the same, fluorescence microscopic analysis was performed using live/dead staining. To a concentration of 100 μg/ml (2 × MBIC), green fluorescence was observed, which divulges the presence of metabolically viable cells. Meanwhile, less number of dead (red fluorescence) cells were observed in HMB-treated (250 and 500 μg/ml) groups, which specifies the non-toxic nature of HMB on HEK-293 cells (Figure 9B).

## Discussion

*Proteus mirabilis* forms extensive crystalline biofilm on indwelling urinary catheters, which blocks urine flow and leads to severe clinical complications. Generally, antibiotic treatment and recurrent catheter changes are ineffective in resolving *P. mirabilis* infections, especially catheter blockages and kidney infections (Sabbuba et al., 2003; Stickler and Jones, 2008). Therefore, there is a need to develop an alternative treatment strategy to combat biofilm-associated *Proteus* infections. To cope with this issue, this study was intended to delve into antibiofilm and antivirulence activity of HMB against *P. mirabilis* crystalline biofilm production. Unlike antimicrobial therapy, the probability of resistance development by pathogens upon treatment with antibiofilm agents is extensively low, as they specifically target virulence factor secretion that ruffles pathogenicity without affecting the metabolic viability of the pathogen (Srinivasan et al., 2018; Martínez et al., 2019).

In the present study, HMB was found to be effective in reducing the biofilm production of *P. mirabilis* ATCC and clinical strain in concentration-dependent manner. HMB showed maximum reduction in biofilm at MBIC (50 μg/ml) with no adverse effects on *P. mirabilis* growth. The results of XTT reduction assay also affirmed the non-bactericidal antibiofilm property of HMB. Further, microscopic analysis also validated the antibiofilm potential of HMB against *P. mirabilis* strains. The biofilm architecture in HMB-treated slides was diminished and poorly developed when compared with that of the untreated control. With this observation, HMB treatment is envisaged to control the biofilm-related infections caused by *P. mirabilis*.

Biofilm-associated bacterial cells are encased in an EPS matrix, which is mainly composed of proteins, polysaccharides, and extracellular DNA (eDNA). These EPS components are essential for bacterial cell adherence, proliferation, and resistance development against antibiotics and host defense system (Flemming and Wingender, 2010). Hence, the effect of HMB on EPS of *P. mirabilis* was examined, which revealed the significant EPS inhibitory potential of HMB at its MBIC. Further, FT-IR analysis divulged the EPS inhibitory potential of HMB (MBIC) upon comparison of IR spectral regions of the components (polysaccharides, protein, and eDNA) of EPS samples extracted from control and HMB-treated *P. mirabilis* culture. The previous report of Issac Abraham et al. (2011) had stated that the antibiofilm efficiency of *Capparis spinosa* fruit extracts against Gram-negative bacterial pathogens was attributable to its ability to inhibit EPS production. Similarly, in the current study, the reduced biofilm production upon HMB treatment is well corroborated with EPS inhibitory potential of HMB. The preformed robust biofilm exhibits decreased susceptibility to host defenses and antimicrobial treatment (de la Fuente-Núñez et al., 2013). Owing to the biofilm forming ability, elimination of *P. mirabilis* from catheterized urinary tract becomes highly challenging. Hence, the present study also focused on analyzing the effect of HMB on preformed biofilm of *P. mirabilis*. The observation of live/dead staining confirmed the dose-dependent killing efficacy of HMB against biofilm-embedded cells.

In addition, motility is a crucial factor for the pathogenicity of *P. mirabilis*, which helps the organism to colonize on biotic and abiotic surfaces through its characteristic flagellum. The results of present study have demonstrated the anti-swarming and anti-swimming efficacy of HMB against *P. mirabilis*. Additionally, HMB modulated the activity of global gene expression regulators that control the expression of virulence factors and swarming of *P. mirabilis*. In this respect, Allison et al. (1993) have also reported that the amino acid glutamine acts as a signaling molecule to initiate the swarming differentiation of *P. mirabilis*. Hence, it is speculated that HMB interferes with transduction of glutamine signals possibly to exert the anti-swarming activity. The in-depth analysis of actual mechanism is under progress. Moreover, the previous report of Foris and Snowden (2017) had stated the association of *P. mirabilis* biofilm formation with its flagella-mediated motility. In this milieu, the anti-swimming and anti-swarming abilities exerted by HMB signify the possibilities of HMB to mitigate the spread of biofilm-associated *P. mirabilis* infections.

Furthermore, the secretion of virulence enzymes, including urease and hemolysin, are most common factors for enriching the pathogenicity of *P. mirabilis* (Schaffer and Pearson, 2015). Among them, urease is liable for struvite and carbonate apatite crystal production through its urea hydrolysis process. The resulting crystal aggregation remains as the primary factor for kidney stone formation and catheter blockage (Jacobsen and Shirtliff, 2011). Further, in the present study, the crystalline nature of *P. mirabilis*-mediated struvite/apatite precipitates was examined by XRD, FT-IR, and FESEM analyses. The results of these morphological examinations and spectroscopy analyses portrayed the degree of crystallization and chemical compositions of the struvite/apatite precipitates. Furthermore, a significant inhibition of urease production upon HMB (MBIC) treatment was observed (Figures 2A,B). Also, the urease activity of *P. mirabilis* causes encrustations and other serious clinical complications in long-term urethral-catheterized patients via crystalline biofilm production that occurred owing to the impact of alkaline pH prevailing in urine (Broomfield et al., 2009). Hence, the current study evaluated the crystalline biofilm inhibitory potential of HMB against *P. mirabilis*. This observation was found to be synchronized with the results of urease inhibitory assay of the present study. These observations led us to envisage that HMB treatment could be able to delay or cease *P. mirabilis*-mediated urinary catheter blockage/encrustation during infections.

On the other hand, hemolysin is a primary cytotoxin, which directly lyses the renal proximal tubular epithelial cells in human (Nagamatsu et al., 2015). Also, the hemolysins were stated to rupture red blood cells often for iron acquisition. Iron is an essential nutrient for microbial proliferation and establishment of infections in host system (Ganz and Nemeth, 2015). HMB was effective in impeding hemolysin production of *P. mirabilis* ATCC and clinical strains (Figure 2A). This might act as a cause for the inability of *P. mirabilis* to survive in the human bloodstream. This prediction was further confirmed through the result of blood survivability assay, wherein the numbers of viable bacterial cells present in HMB-treated blood serum were reduced when compared with those of the untreated control. In addition, HMB treatment reduced the production of siderophore, an iron acquisition molecule. Owing to the HMB’s siderophore inhibitory activity, the iron deficiency that occurs in the iron-stressed urinary tract environment is envisaged to affect the *P. mirabilis* colonization and its pathogenicity during infection.

To clarify the plausible molecular mechanism demonstrated by HMB on *P. mirabilis*, transcriptomic analysis of candidate genes, which directly or indirectly encodes the biofilm and other virulence factors, was examined. The relative expression level of *P. mirabilis* virulence traits upon HMB (MBIC) treatment is shown in Figure 8, wherein the genes *flhD* and *flhB* are responsible for the regulation of flagella-mediated motility of *P. mirabilis*. The swarming behavior of *P. mirabilis* on solid surface is enacted by its flagellum (Patrick and Kearns, 2012). During swarming, the expression of flagellar biosynthesis genes is coordinately regulated in a hierarchy. The flagellar heteromeric complex with master transcriptional regulator *flhD* regulates the flagellum assembly (like decorating the basal body) by binding to *flhB* promoter region and leads to the biogenesis of flagellar proteins expression, which in turn harvests the energy for flagellar rotation (Claret and Hughes, 2000; Patrick and Kearns, 2012). In the present study, the qRT-PCR analysis reveals that HMB treatment significantly downregulated the expression level of *flhD* and *flhB* genes, which correlated well with the observation of *in vitro* swarming assay.

And, the gene *speA* (encodes arginine decarboxylase) is also responsible for *P. mirabilis* swimming and swarming motility, which helps flagellar rotation fueled by proton motive force through utilization of intracellular protons (Armbruster et al., 2014). The gene expression analysis revealed that the expression level of *speA* gene was considerably downregulated upon HMB treatment (Figure 8). On the contrary, the gene *rsbA* acts as a negative regulator for *P. mirabilis* swarming migration (Liaw et al., 2001). Wang et al. (2006) had reported that resveratrol inhibited the swarming motility of *P. mirabilis* through an RsbA-dependent pathway. Figure 8 shows no significant relative fold difference in expression of *rsbA* gene under HMB treatment compared with the control. This observation indicates that HMB’s anti-swarming activity is not dependent on the RsbA pathway in *P. mirabilis*.

Further, the expression level of virulence genes upon HMB treatment was evaluated to validate the *in vitro* bioassay results. Urease, the primary virulence enzyme production in *P. mirabilis*, was regulated by the transcriptional regulator UreR. This positively activates the expression of urease operon (*ure* gene cluster), wherein the gene *ureR* acts as a transcription initiation codon (Dattelbaum et al., 2003). In this concern, the present study reveals that HMB treatment significantly inhibits the urease production through downregulation of *ureR* gene expression. Likewise, the genes *hpmA* and *hpmB* are responsible for *P. mirabilis* hemolysin production (Cestari et al., 2013). HpmA hemolysin is activated when its N-terminal peptide is cleaved, resulting in tissue damage; and HpmB is responsible for HpmA activation and transport. The expression level of *hpmA* and *hpmB* genes was also found to be downregulated upon HMB treatment. These observations were found to comply with the urease and hemolysin inhibitory abilities of HMB observed in the *in vitro* assays.

Moreover, the non-cytotoxic potential of the multifunctional phytocompound HMB was explicated using human kidney cells. The obtained results showed that HMB did not affect the viability of HEK-293 cells even up to 100 μg/ml of concentration. This observation revealed the innocuous nature of HMB, which concludes that HMB could be used as a potential therapeutic and preventive agent for *P. mirabilis* infections.

## Conclusion

Based on the present study, it could be concluded that HMB treatment efficiently inhibited the pathogenicity of *P. mirabilis* through downregulation of expression level of virulence genes involved in flagellar motility, biofilm formation, EPS production, biosynthesis of urease, and hemolysin enzymes. These results are also reflected in the *in vitro* virulence assays. Owing to this virulence targeted therapeutic activity, the resistance development in *P. mirabilis* to HMB is presumed to be very scarce. Furthermore, HMB did not exhibit any cytotoxicity against human kidney cell lines. Hence, treatment with HMB as an antibiofilm/antivirulence agent could be a promising therapy to prevent urinary catheter blockage and other biofilm-associated *P. mirabilis* infections.

## Data Availability Statement

All datasets generated for this study are included in the article/Supplementary Material.

## Ethics Statement

The studies involving human participants were reviewed and approved by the Institutional Ethics Committee, Alagappa University, Karaikudi. Written informed consent for participation was not required for this study in accordance with the national legislation and the institutional requirements.

## Author Contributions

RD designed the study and wrote the manuscript. RD, GA, KN, and PK performed the experiments. AV provided materials and reagents. SP provided the test pathogen clinical strain. RD and RA interpreted the data. All authors approved the final version of the manuscript.

## Conflict of Interest

The authors declare that the research was conducted in the absence of any commercial or financial relationships that could be construed as a potential conflict of interest.
