# Supplementary material for: Explication of the Potential of 2-Hydroxy-4-Methoxybenzaldehyde in Hampering Uropathogenic Proteus mirabilis Crystalline Biofilm and Virulence
Source: Front Microbiol. 2019 Dec 10;10:2804. doi: 10.3389/fmicb.2019.02804 (PMC6914683; doi:10.3389/fmicb.2019.02804)
Supplement: Supplementary file 1 [file Data_Sheet_1.pdf]

Supplementary Material

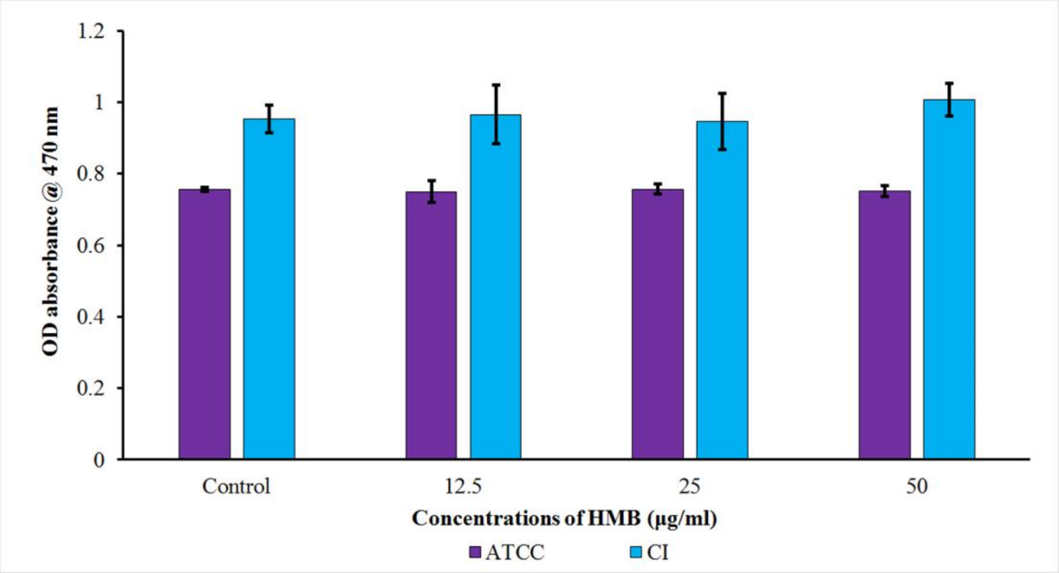

Figure S1 Effect of HMB on metabolic viability of *P. mirabilis* ATCC and CI strains.

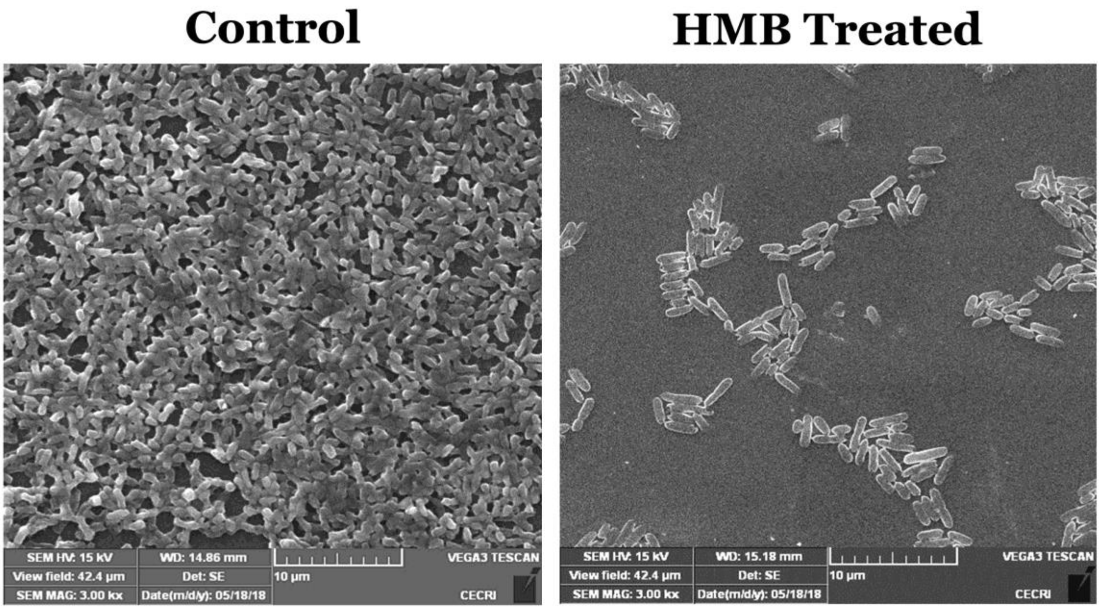

Figure S2 FESEM analysis of *P. mirabilis* biofilm under HMB treatment at MBIC (scale bar–10 µm)
